# Bioenergetics of the VO_2_ slow component between exercise intensity domains

**DOI:** 10.1007/s00424-020-02437-7

**Published:** 2020-07-14

**Authors:** Alessandro L. Colosio, Kevin Caen, Jan G. Bourgois, Jan Boone, Silvia Pogliaghi

**Affiliations:** 1 Department of Neurosciences, Biomedicine and Movement Sciences, University of Verona, Via Casorati 43, 37131 Verona, Italy; 2 Department of Movement and Sports Sciences, Ghent University, Watersportlaan 2, Ghent, Belgium

**Keywords:** Oxidative metabolism, VO2 kinetics, Exercise physiology, Oxygen consumption, Excess VO2, Loss of efficiency

## Abstract

During heavy and severe constant-load exercise, VO_2_ displays a slow component (VO_2sc_) typically interpreted as a loss of efficiency of locomotion. In the ongoing debate on the underpinnings of the VO_2sc_, recent studies suggested that VO_2sc_ could be attributed to a prolonged shift in energetic sources rather than loss of efficiency. We tested the hypothesis that the total cost of cycling, accounting for aerobic and anaerobic energy sources, is affected by time during metabolic transitions in different intensity domains. Eight active men performed 3 constant load trials of 3, 6, and 9 min in the moderate, heavy, and severe domains (i.e., respectively below, between, and above the two ventilatory thresholds). VO_2_, VO_2_ of ventilation and lactate accumulation ([La^−^]) were quantified to calculate the adjusted oxygen cost of exercise (AdjO_2Eq_, i.e., measured VO_2_ − VO_2_ of ventilation + VO_2_ equivalent of [La^−^]) for the 0–3, 3–6, and 6–9 time segments at each intensity, and compared by a two-way RM-ANOVA (time × intensity). After the transient phase, AdjO_2Eq_ was unaffected by time in moderate (ml*3 min^−1^ at 0–3, 0–6, 0–9 min: 2126 ± 939 < 2687 ± 1036, 2731 ± 1035) and heavy (4278 ± 1074 < 5121 ± 1268, 5225 ± 1123) while a significant effect of time was detected in the severe only (5863 ± 1413 < 7061 ± 1516 < 7372 ± 1443). The emergence of the VO_2sc_ was explained by a prolonged shift between aerobic and anaerobic energy sources in heavy (VO_2_ − VO_2_ of ventilation: ml*3 min^−1^ at 0–3, 0–6, 0–9 min: 3769 ± 1128 < 4938 ± 1256, 5091 ± 1123, [La^−^]: 452 ± 254 < 128 ± 169, 79 ± 135), while a prolonged metabolic shift and a true loss of efficiency explained the emergence of the VO_2sc_ in severe.

**Electronic supplementary material:**

The online version of this article (10.1007/s00424-020-02437-7) contains supplementary material, which is available to authorized users.

## Introduction

After the cardio-dynamic phase, oxygen consumption (VO_2_) during constant heavy and severe intensity exercise, is better fitted by a two-component rather than a single-component model. Based on this observation, it has been suggested, that a slow component of VO_2_ exists (VO_2sc_), that does not start at exercise onset but rather appears later in exercise (time delay ~ 120–180 s) [9]. Furthermore, it has been assumed, but not proven, that in association with VO_2sc_, the muscle displays an increasing energy demand as a function of time [10].

VO_2sc_ represents an increased O_2_ cost of locomotion when exercise is protracted more than 3 min at a constant workload above the lactate threshold (LT) [9]. Typically, when exercise is performed between LT and the critical power, (i.e. heavy intensity domain) VO_2sc_ tends to a delayed steady-state (i.e. compared to moderate). On the contrary, when effort rises above critical power (i.e. severe exercise domain) a steady-state is not achievable and VO_2_ increases tending to the maximal oxygen consumption (VO_2max_) [9]. The magnitude of VO_2sc_ is considered to be associated with exercise intolerance and fatigue [7], and the study of this phenomenon is particularly important in diseased/frail populations (e.g. [19, 24]). Nevertheless, despite the efforts of the past 40 years to clarify the exact physiological underpinnings of the VO_2sc_, these remain debated even in healthy subjects. [9].

A key finding from the past studies was that roughly 85% of the VO_2sc_ originates from the contracting muscles, while the remaining 15% corresponds to the increased VO_2_ cost of ventilation [20]. Focusing on the muscular component of the VO_2sc_, subsequent investigations proposed that the recruitment of less efficient type II fibres necessary to maintain a specific power output (PO) [9] or metabolic instability due to metabolites accumulation occurring within the working fibres [26, 28] could elicit an increased cost of locomotion. Nevertheless, a satisfactory theory explaining the mechanisms underpinning the VO_2sc_ is still missing.

In this context, a recent study [17] refuted that the energy demand of a constant, high-intensity exercise changes over time. By subtracting the VO_2_ cost of ventilation and accounting for the contribution of the glycolytic energy sources [17, 21, 22] O’Connell et al. quantified the total, adjusted metabolic cost of muscle exercise over time during a constant load trial in the severe domain. The authors concluded that the overall cost of locomotion does not increase over time, other than what required by the augmenting cost of ventilation. The apparent VO_2_ increases over time would be the result of the prolonged shift in metabolic sources beyond the first 3 min of exercise (i.e. an increased contribution of the aerobic metabolism to ATP resynthesis, mirrored by a decreased contribution of anaerobic ATP resynthesis over time)_._ In other words, these findings suggest that VO_2sc_ may in fact not represent a true loss of efficiency as a function of time but rather a prolonged adjustment of the oxidative metabolism. However, while this new hypothesis could explain why a satisfying explanatory theory of VO_2sc_ is still missing, the amount of literature that reported VO_2sc_ in different exercise modalities (i.e. whole-body vs isolated muscles) and models (i.e. humans vs animals) calls for further investigation [9]. In particular, the intensity domain in which O’Connell measures were obtained is not fully clear; while most studies in the field of VO_2_ kinetics define intensity domains based on gas exchange thresholds, O’Connell used lactate-based measures. Yet, the correspondence between the above methods remains a controversial issue [3, 12, 13]. In addition, loss of efficiency, the metabolic shift among substrates and excess ventilation manifest and increase differently over time in the three domains of exercise (e.g. no loss of efficiency is present in the moderate domain while an elevated steady state is reached in the heavy yet not in the severe domain) [10]. Therefore, O’Connell’s findings need to be confirmed and broadened with particular consideration to the three intensity domains of exercise.

Accordingly, by calculating the energy cost of ventilation, the glycolytic contribution to exercise, and directly measuring the aerobic cost of locomotion over time, we tested if and to what extent a true loss of efficiency during cycling explains the emergence of the slow component of VO_2_ in different intensity domains. Specifically, we hypothesised that (i) the overall cost of locomotion would not be affected by the time during metabolic transitions in the moderate and heavy exercise domains (i.e. no loss of efficiency over time exists at these intensities but rather a prolonged metabolic shift is responsible for the observed VO_2sc_); (ii) the overall cost of locomotion would be affected by the time during metabolic transitions in the severe exercise domain (i.e. a prolonged metabolic shift is not sufficient to explain the emerge loss the observed VO_2sc_ and a true loss of efficiency over time will manifest at this intensity).

## Methods

### Ethical approval

The study was conducted according to the Declaration of Helsinki and all procedures were approved by the University of Verona Ethics Committee for Research on Human Subjects (CARU no 15/2019). Procedures and risks were explained to each subject, and all participants volunteered and gave informed written consent to participate before the start of the study.

### Participants

Eight active men were recruited in the study (age 25 ± 2 years, body mass 74 ± 10 kg, height 181 ± 5 cm, VO_2max_ 49.3 ± 3.4 ml*kg^−1^*min^−1^, performing recreational aerobic activity 3–4 times × week). Inclusion criteria were male sex and age between 20 and 35 years; exclusion criteria were smoking and any condition that could influence the physiological responses during testing. All participants were instructed to avoid caffeine consumption and physical activity respectively for at least 8 h and 24 h before each testing session. Moreover, they received a standard and individualized food intake prescription before all the testing sessions to minimize variability of glycogen stores and glucose oxidation (i.e. 2 g of low glycemic index carbohydrates per kg of body weight, 2 h before testing; 0.5 L of water in the 90 min before testing; restriction from caffeine during the 8 h before testing).

### Experimental protocol

After medical clearance, participants visited the laboratory on ten occasions within a maximum of 4 weeks. All subjects completed: (i) a preliminary maximal ramp incremental exercise test to exhaustion for the determination of gas exchange threshold (GET), respiratory compensation point (RCP) and the peak of oxygen uptake (VO_2peak_); (ii) three constant load trials (CLT) respectively of 3, 6 and 9 min in the “moderate” exercise intensity domain (iii) three CLT respectively of 3, 6 and 9 min in the “heavy” exercise intensity domain (iv) three CLT trials respectively of 3, 6 and 9 min in the “severe” exercise intensity domain. Tests of (ii), (iii) and (iv) aimed at determining VO_2_ response and blood lactate ([La^−^]) accumulation as a function of time in the three exercise intensity domains in order to evaluate the overall energetic contribution of the aerobic and anaerobic metabolisms [22] to ATP turnover. Moreover, these tests were executed in randomized order with the only exception of the longest CLT in the “severe” exercise domain, that was completed as first to assure that subjects were able to sustain the PO for the required time. All exercise tests were conducted on an electromagnetically braked cycle ergometer (Sport Excalibur, Lode, Groningen, Netherlands), at a similar time of the day in an environmentally controlled laboratory (18 °C, 55–65% relative humidity).

### Ramp incremental test

The ramp incremental test consisted of a 3-min baseline cycling at 50 W, followed by a 30-W*min^−1^ increase in PO until volitional exhaustion. Participants were asked to pick a self-selected cadence in the range of 70–90 rpm and to maintain it throughout all subsequent tests. Failure to maintain the indicated cadence within 5 rpm (for longer than 5 s) during testing despite strong verbal encouragement was considered as the criterion for exhaustion. Breath-by-breath pulmonary gas exchange and ventilation were continuously measured using a metabolic cart (Jaeger Oxycon Pro, Viasys Healtcare GmbH, Höchberg, Germany) as previously described [23]. Heart rate (HR) was monitored continuously (H7 Sensor, Polar, Kempele, Finland).

### Constant load trials

After the preliminary ramp incremental test, subjects completed 3 CLT within each exercise intensity domain (i.e. moderate, heavy, and severe [18]) in a randomized order:

i) Moderate: 3 CLT respectively of 3,6 and 9 min at 80% of GET.
ii) Heavy: 3 CLT respectively of 3,6 and 9 min at 50%Δ between GET and RCP.
iii) Severe: 3 CLT respectively of 3,6 and 9 min at 60%Δ between GET and VO_2peak_.

Each CLT was preceded by a 3-min warm-up at 20 W. Throughout the test, subjects kept the same, constant rpm and bike position as selected during the ramp incremental test.

VO_2_ and HR data were measured with the same method described for the ramp incremental test. Moreover, capillary blood samples (65 μl) were drawn from the fingertip in the last 30 s of warm-up and at the 1st, 3rd, 5th, and 7th min after each test and were immediately analysed (Radiometer ABL90 FLEX, Radiometer Medical ApS, Brønshøj, Denmark) to measure [La^−^]. The highest value was considered as the peak of blood lactate concentration and used for further analysis to calculate anaerobic energetic contribution.

### Data analysis

#### Ramp incremental test

For the gas exchange variables, aberrant data-points that lay 3 SD from the local mean were removed, and trials were linearly interpolated on a 1-s basis and then averaged every 5 s. VO_2peak_ was determined as the highest VO_2_ obtained over a 10-s interval [6]. GET and RCP were determined with the standard technique from gas exchange variables by three blinded expert reviewers as detailed elsewhere [6]. Briefly, GET was determined by visual inspection as the VO_2_ at which CO_2_ output began to increase out of proportion in relation to VO_2_, with a systematic rise in the ventilation (VE)-to-VO_2_ relation and end-tidal PO_2_ whereas the ventilatory equivalent of VCO_2_ (VE/VCO_2_) and end-tidal PCO_2_ is stable [2]. RCP was determined as the point where end-tidal PCO_2_ began to fall after a period of isocapnic buffering [27]. This point was confirmed by examining VE/VCO_2_ plotted against VO_2_ and by identifying the sec breakpoint in the VE-to-VO_2_ relation. Finally, we determined the constant workload equivalent to the specific moderate (80% of GET), heavy (50% Δ between GET and RCP), and severe (60%Δ between GET and VO_2peak_) VO_2_ targets. To this aim, the VO_2_/W relationship identified with the incremental test was left-shifted to account for the mean response time [6].

#### Constant load trials

VO_2_, VCO_2_, and VE during CLT were sampled breath-by-breath and interpolated using the same procedure described for the ramp incremental test. Interpolated data from different CLT performed at the same exercise intensity were mediated in order to reduce breath-by-breath signals variability (data from 3 tests were mediated in the time segment from 0 to 3 min and data from 2 tests were mediated between 3 and 6 min) [11]. The sum of the oxygen consumed during each 3-min time segment was then considered as the aerobic energetic contribution to exercise.

VO_2sc_ was calculated as the sum of the amount of oxygen exciding the VO_2_ reached at the end of the 0-to-3-min time segment [25].

Work of breathing (WB) was calculated based on VE using the equation by Coast et al.:

WB = − 0.430 + 0.050 * VE + 0.00161 VE^2^

Then, the WB was used to calculate the amount of VO_2_ requested by ventilatory muscles (VO_2VM_):

VO_2VM_ = 34.9 + 7.45 *WB [4]

Anaerobic (glycolytic) contribution to exercise was calculated from the amount of [La^−^] accumulation over time calculated as follows:

- 0–3 min segment [La^−^] accumulation = 3-min CLT peak [La^−^] − warm-up [La^−^]
- 3–6 min segment [La^−^] accumulation = 6-min CLT peak [La^−^] − 3-min CLT peak [La^−^]
- 6–9 min segment [La^−^] accumulation = 9-min CLT peak [La^−^] − 6-min CLT peak [La^−^]

The so obtained values were utilized to estimate the energy contribution from anaerobic glycolysis in each time segment, based on the oxygen equivalent for lactate of Di Prampero (i.e. 1 mmol*L^−1^ [La^−^] accumulation = 3.0 ml*kg^−1^ VO_2_) [22].

Overall energetic cost of the activity (expressed as ml of oxygen) was calculated as described by O’Connell et al. and defined as “adjusted oxygen equivalent” (AdjO_2Eq_): o.

$$ {\mathrm{AdjO}}_{2\mathrm{Eq}}=\mathrm{measured}\ {\mathrm{VO}}_2\ \left(\mathrm{ml}\ {\mathrm{O}}_2\ast 3\ {\min}^{-1}\right)-{\mathrm{VO}}_{2\mathrm{VM}}\ \left(\mathrm{ml}\ {\mathrm{O}}_2\ast 3\ {\min}^{-1}\right)+\mathrm{Oxygen}\ \mathrm{Equivalent}\ \mathrm{of}\ \mathrm{Lactate}\ \left(\mathrm{ml}\ {\mathrm{O}}_2\ast 3\ {\min}^{-1}\right) $$

VO_2_ gain (VO_2gain_, i.e. the amount of oxygen equivalent utilized to sustain each *W* during cycling) was calculated as the ratio between the amount of AdjO_2Eq_ required to sustain exercise during a specific time segment and the number of W imposed by the test: VO_2gain_ = (3 min AdjO_2Eq_ − warm-up AdjO_2Eq_) / (test PO (*W*) − warm-up PO (*W*)).

### Statistics

After assumptions verification (i.e., normality, homogeneity of variance), the within-subject coefficient of variation and a two-way repeated-measure ANOVA (trial × intensity domain) were used to evaluate VO_2_ data repeatability measured at the end of the third min of exercise of each CLT.

Two-way repeated-measure ANOVA was also performed to assess differences over time between different intensities domains (time segment × intensity domain) for VO_2_, VO_2VM_, O_2_ equivalent of [La^−^], AdjO_2Eq_, VO_2gain_. The amplitude of the VO_2sc_ in different intensity domains was compared using a one-way repeated measures ANOVA (intensity domain). Post-hoc analyses were performed using the Holm-Sidak method. Moreover, partial eta squares (η_p_^2^) were calculated to quantify the effects sizes of different independent variables during the constant load trials [14]. Based on an expected standard deviation of breath-by-breath VO_2_ measurements for steady-state exercise equal to 2.5%, and a minimum detectable change in VO_2_ of 100–170 ml·min^−1^ at a VO_2_ of 2.1 to 3.5 L·min^−1^ [12], the minimum sample size to obtain a power of 0.8 was 6 individuals.

Data are presented as means ± SD. All statistical analyses were performed using Sigmaplot version 12 and *α* was set in advance at the 0.05 level. Statistical significance was accepted when *p* < α.

## Results

Subjects’ anthropometrical and functional characteristics obtained during the ramp incremental test are reported in Table 1.

**Table 1:** Subjects’ anthropometrical and functional characteristics obtained during the ramp incremental test

Subjects characteristics				Ramp incremental test					
**#**	Weight (kg)	Height (cm)	Age (years)	PPO (W)	VO_2peak_ (ml*min^−1^)	VO_2peak_ (ml*min^−1^*kg)	MRT (sec)	GET (ml*min^−1^)	RCP (ml*min^−1^)
8 ♂	74 ± 9	180 ± 5	25 ± 1	376 ± 36	3643 ± 457	49 ± 3	41 ± 12	2418 ± 385	3093 ± 377

Values are means ±SD. *PPO*, peak PO; *VO*_2peak_: peak of oxygen consumption; *MRT*, mean response time; *GET*, gas exchange threshold; *RCP*, respiratory compensation point

**Fig. 1:**
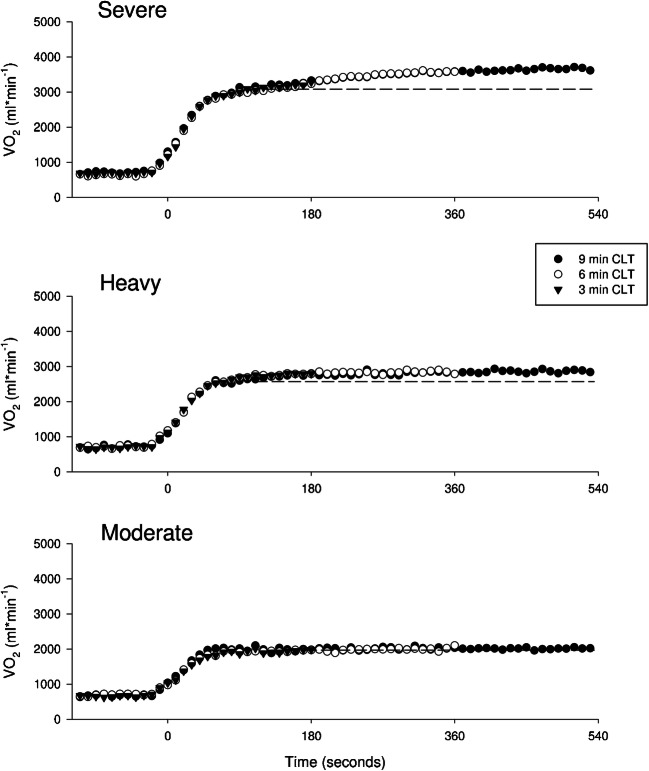
Repeatability of VO_2_: group mean VO_2_ data are displayed as 10s-averages respectively for the “severe” (top panel), “heavy” (medium panel), and “moderate” (bottom panel) exercise intensity domain. Symbols represent the three duration trials: black dots = 9-min CLT, white dots = 6-min CLT, black triangles = 3-min CLT

Repeatability of group mean VO_2_ data (10-s averages) is displayed in Fig. 1. Average measured VO_2_ displays a complete overlap among the three durations trials (3, 6, 9 min) at the three intensities (moderate, heavy, severe). Furthermore, mean VO_2_ values of the last 10 s of the third min of exercise for the 9, 6, and 3-min CLT were respectively: 3328 ± 470, 3231 ± 434, 3285 ± 443 ml*min^−1^ (severe) 2804 ± 408, 2783 ± 492, 2793 ± 470 ml*min^−1^ (heavy), and 1979 ± 281, 1966 ± 330, 2095 ± 258 ml*min^−1^ (moderate), with a mean within-subject coefficient of variation of 3.3 ± 2.4% (severe), 4.3 ± 1.4% (heavy), 3.7 ± 1.4% (moderate). As expected, ANOVA on these VO_2_ values revealed a significant main effect of the intensity domain at which exercise was performed (*p* ≤ 0.001, *η*_p_^2^: 0.95) but no significant main effect among the three trials performed at the same intensity (*p* = 0.437, *η*_p_^2^: 0.08).

Mean values of PO and per-min measures of VO_2_, VCO_2_, VE, and HR in the last 30 s of each time segment are displayed in Table 2, along with measures of [La^−^] at the end of the warm-up and at the end of each time segment (peak [La^−^]). ANOVA revealed a significant “time segment” × “intensity domain” interaction for VO_2_ (*p* ≤ 0.001, *η*_p_^2^: 0.57), VCO_2_ (*p* = 0.014, *η*_p_^2^: 0.35), VE (*p* ≤ 0.001, *η*_p_^2^: 0.73), HR (*p* = 0.011, *η*_p_^2^: 0.30), and [La^−^] (*p* ≤ 0.001, *η*_p_^2^: 0.29). In detail, HR and [La^−^] reached a steady-state within the 3rd min only in the moderate and heavy-intensity exercise trials, while they increased over time in the severe exercise trials. VO_2_, VCO_2_, and VE reached a steady-state within the 3rd min in the moderate-intensity and within the 6th min of the heavy-intensity exercise trials. On the contrary, these parameters continued to increase significantly over time in the severe exercise trials. Finally, a significant main effect of “intensity” on the magnitude of VO_2sc_ was detected (*p* ≤ 0.001, *η*_p_^2^: 0.77), and VO_2sc_ amplitude was increasing going from moderate (12.7 ± 45.6 ml*min^−1^) to heavy (136.7 ± 99.3 ml*min^−1^) and severe (416.7 ± 165.9 ml*min^−1^; post-hoc: moderate vs heavy *p* = 0.026 moderate vs severe *p* ≤ 0.001, heavy vs severe *p* = 0.021).

**Table 2:** Metabolic response in the moderate, heavy and severe exercise domains

	Load	VO_2_	VCO_2_	VE	HR	[La^−^]
Time Segment	(W)	(ml*min^−1^)	(ml*min^−1^)	(L*min^−1^)	(b*min^−1^)	(mmol*L)
Severe						
Warm-up	20.0 ± 0.0	1034.5 ± 51.4	743.8 ± 479.1	17.7 ± 2.2	73.2 ± 9.6	1.7 ± 0.6
0 to 3 min	267.0 ± 37.5	3198.7 ± 435.6	3585.8 ± 599.2	92.9 ± 26.9	157.3 ± 4.7	6.7 ± 1.6
3 to 6 min	267.0 ± 37.5 **#**	3489.1 ± 498.8 **#**	3792.3 ± 597.9 **#**	109.9 ± 31.1 **#**	169.5 ± 5.5	9.2 ± 2.4 **#**
6 to 9 min	267.0 ± 37.5 #*	3615.5 ± 521.9 #*	3888.1 ± 634.2 #*	119.1 ± 31.0 #*	175.2 ± 4.8 **#**	10.7 ± 2.5 #*
Heavy						
Warm-up	20.0 ± 0.0	1067.7 ± 57.6	605.8 ± 58.6	18.6 ± 2.6	74.2 ± 8.4	1.8 ± 0.8
0 to 3 min	208.9 ± 28.7	2771.6 ± 429.3	2748.8 ± 438.2	66.5 ± 14.4	141.5 ± 11.2	3.4 ± 1.1
3 to 6 min	208.9 ± 28.7 **#**	2851.5 ± 425.4 **#**	2818.3 ± 400.8 **#**	70.9 ± 13.4 **#**	146.3 ± 15.3	3.9 ± 1.4
6 to 9 min	208.9 ± 28.7 **#**	2822.7 ± 395.7 **#**	2741.1 ± 367.5 **#**	71.0 ± 13.1 **#**	151.1 ± 15.2	4.1 ± 1.4
Moderate						
Warm-up	20.0 ± 0.0	1026.3 ± 65.3	566.4 ± 60.7	17.1 ± 1.7	73.3 ± 8.5	1.7 ± 0.6
0 to 3 min	128.7 ± 27.0	1951.6 ± 261.7	1731.0 ± 275.5	42.9 ± 8.1	117.5 ± 23.6	2.4 ± 0.8
3 to 6 min	128.7 ± 27.0	1987.1 ± 277.4	1841.5 ± 275	45.3 ± 7.2	122.0 ± 27.0	1.8 ± 0.6
6 to 9 min	128.7 ± 27.0	1992.3 ± 298.7	1871.7 ± 332.7	47.6 ± 9.8	122.9 ± 29.0	1.3 ± 0.3

Values are means ±SD. Values of PO and per-min measures of VO_2_, VCO_2_, VE, and HR in the last 30s of each time segment are displayed, along with measures of lactate concentration ([La^−^]) at the end of warm-up and at the end of each time segment. ANOVA revealed a significant “intensity” × “time” interaction for VO_2_ (*p* ≤ 0.001), VCO_2_ (*p* = 0.014), VE (*p* ≤ 0.001), HR (*p* = 0.011) and [La^−^] (*p* ≤ 0.001). Multiple comparisons are also displayed: # represents significant statistical difference with 0 to 3 min segment; * represents significant statistical difference with 3 to 6 min segment. For greater clarity were omitted: comparisons vs warm-up (always significantly different, with the only exception of [La^−^] in the moderate exercise intensity domain) and between exercise domains (always significantly different, with the only exception of HR between “severe” and “heavy” during the 0–3 segment)

**Fig. 2:**
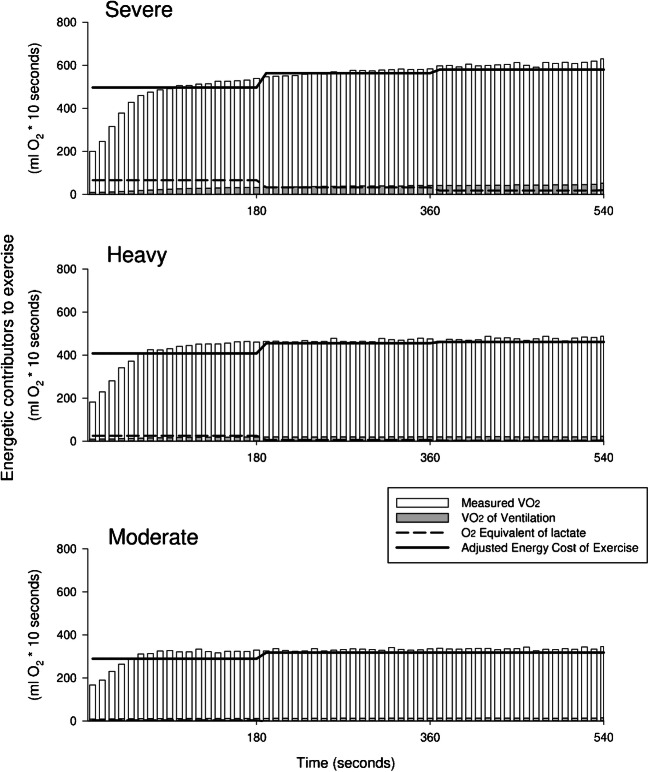
An overview of the energetic contributors to exercise is reported in 10-s averages for the “severe” (top panel), “heavy” (medium panel), and “moderate” (bottom panel) exercise intensity domain. White columns represent directly measured VO_2_. Grey columns indicate the O_2_ cost requested by ventilation. The black dashed line displays the energy provided by glycolytic sources over 3 min segments. Finally, the black solid line represents the adjusted cost of exercise accounting for both aerobic and glycolytic energy sources. Please note that during all the first 3 min segments, the contribution of immediate energy sources (O_2_ and phosphocreatine) was not accounted and was probably the cause of the lower adjusted VO_2_ between the first and the successive time segments [8, 22]

**Fig. 3:**
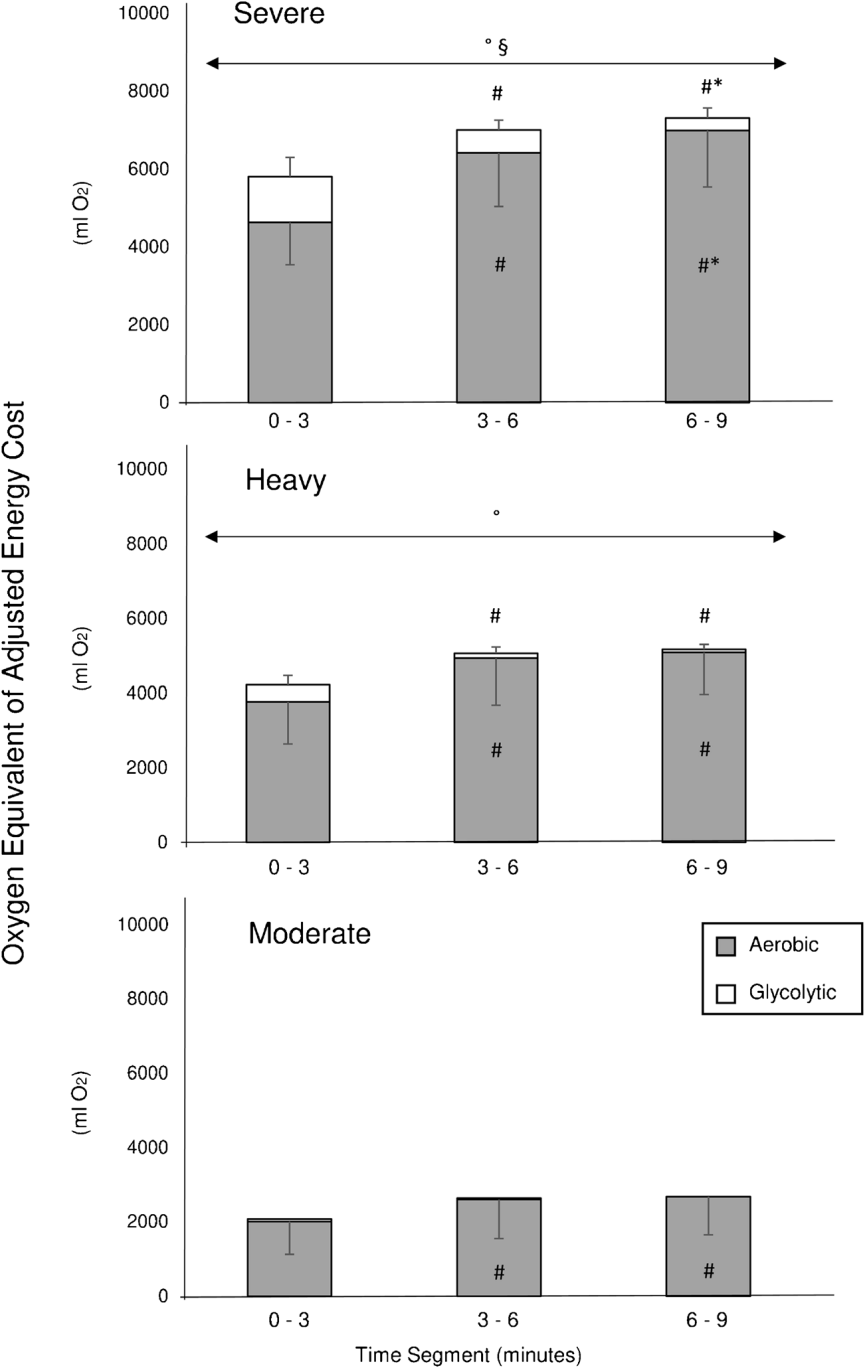
Three-min mean oxygen equivalents of aerobic (grey, i.e. VO_2_ subtracted by VO_2VM_) and glycolytic (white, i.e. [La^−^] equivalent) cost of exercise are represented respectively for the “severe” (top panel), “heavy” (medium panel) and “moderate” (bottom panel) exercise intensity domain. ANOVA revealed a significant “intensity” × “time” interaction both for aerobic (*p* ≤ 0.001, *η*_p_^2^: 0.81) and anaerobic (*p* ≤ 0.001, *η*_p_^2^: 0.58) cost of exercise. Multiple comparisons are displayed as: # significant statistical difference with 0 to 3 min segment; * significant statistical difference with 3 to 6 min segment. ° and § represent respectively statistical difference with the “moderate” and “heavy” exercise intensity domain. Please note that values measured during warm-up were subtracted

An overview of the energetic contributors to exercise (i.e., measured VO_2_, VO_2VM_, [La^−^] equivalent of O_2_, and AdjO_2Eq_) is reported as 10-s averages and over 3-min time segments in Fig. 2 and in Table 3. For all time segments, the contribution of anaerobic glycolysis (as represented by [La^−^] equivalent) was significantly increased going from moderate to heavy to severe intensity trails (significant main effect of intensity, *p* ≤ 0.001, *η*_p_^2^: 0.91). Furthermore, the contribution of anaerobic glycolysis in the three domains was significantly affected by time (significant main effect of time segment *p* ≤ 0.001, *η*_p_^2^: 0.67). In particular, the post-hoc analysis revealed a limited and unvaried contribution of anaerobic glycolysis for the moderate-intensity domain (0–3 vs 3–6 time segments *p* = 0.772, 3–6 vs 6–9 *p* = 0.947); in heavy, a decreased contribution of anaerobic glycolysis from 0 to 3 to 3–6 time segments, yet no further after that (0–3 vs 3–6 *p* = 0.014, 3–6 vs 6–9 *p* = 0.429); finally, a continuous decrease in the contribution of anaerobic glycolysis over time in the severe exercise trials (0–3 vs 3–6 p ≤ 0.001, 3–6 vs 6–9 *p* = 0.024; Fig. 3).

**Table 3:** Energetic contributors to exercise in the moderate, heavy and severe exercise domains

Time segment	VO_2_ (ml O_2_*3min^−1^)	VO_2VM_ (ml O_2_*3min^−1^)	[La^−^] Equivalent (ml O_2_*3min^−1^)	AdjO_2Eq_ (ml O_2_*3min^−1^)	VO_2gain_ (ml O_2_*min^−1^ * W)
Severe					
0 to 3 min	5037 ± 1155	356 ± 113	1182 ± 492	5863 ± 1413	7.9 ± 1.2
3 to 6 min	7096 ± 1509 #	623 ± 234 #	588 ± 256 #	7061 ± 1516 #	9.5 ± 1.2 #
6 to 9 min	7780 ± 1573 #*	729 ± 270 #*	321 ± 259 #*	7372 ± 1443 #*	9.9 ± 1.1 #
Heavy					
0 to 3 min	4062 ± 1184	216 ± 62	452 ± 254	4278 ± 1074	7.5 ± 1.1
3 to 6 min	5289 ± 1321 #	295 ± 77 #	128 ± 169 #	5121 ± 1268 #	9.0 ± 1.3 #
6 to 9 min	5450 ± 1180 #	304 ± 77 #	79 ± 135 #	5225 ± 1123 #	9.2 ± 1.0 #
Moderate					
0 to 3 min	2200 ± 906	140 ± 25	67 ± 94	2126 ± 939	6.4 ± 1.8
3 to 6 min	2823 ± 1083 #	169 ± 33	34 ± 58	2687 ± 1036 #	8.1 ± 1.7 #
6 to 9 min	2910 ± 1067 #	189 ± 40	0 ± 0	2731 ± 1035 #	8.3 ± 1.8 #

Values are means ±SD. Values are reported over 3-min time segments. VO_2_: directly measured VO_2_; VO_2VM_: VO_2_ requested by ventilatory muscles; [La^−^] equivalent: oxygen equivalent of lactate; AdjO_2Eq_: energy cost of exercise expressed as a sum of aerobic and glycolytic sources (VO_2_ + [La^−^] equivalent) and subtracted by VO_2VM_. ANOVA revealed a significant “intensity” × “time” interaction for VO_2_ (*p* ≤ 0.001), VO_2VM_ (*p* ≤ 0.001), [La^−^] equivalent (*p* ≤ 0.001), and AdjO_2Eq_ (*p* ≤ 0.001). Multiple comparisons are displayed in the table: # represents significant statistical difference with 0 to 3 min segment; * represents significant statistical difference with 3 to 6 min segment. Please note that values measured during warm-up were subtracted. For greater clarity comparisons between intensity domains were omitted (always significantly different, with the exceptions of (i) [La^−^] equivalent between “heavy” and “moderate” during the 3–6 and 6–9 time segments and (ii) VO_2gain_ between “severe” and “heavy” in the 0–3 segment (iii) VO_2gain_ between “severe” and “heavy” and “heavy” and “moderate” in the 3–6 segment)

## Discussion

By comprehensively quantifying aerobic and glycolytic energy sources used during exercise, deprived of the VO_2_ cost of ventilation, this study tested the hypothesis that the overall cost of cycling (i.e. AdjO_2Eq_) is affected by the time during metabolic transitions in the moderate, heavy and severe intensity domains. In agreement with our hypothesis, our findings suggest that (i) the overall cost of locomotion does not increase over time in the moderate domain after the 3rd min of exercise; (ii) in the heavy intensity domain, the emergence of a VO_2_ slow component is ascribable to a “metabolic shift” between aerobic and anaerobic metabolisms that protracted beyond the 3rd min of exercise and to a small yet significant increase in the VO_2_ of ventilation, rather than to an actual increased cost of locomotion after the 3rd min; (iii) finally, only in the severe domain the raise of the VO_2_ slow component deprived of its ventilatory part was not completely explained by a prolonged metabolic shift between energy sources, and the further increase in AdjO_2Eq_ was possibly indicating that a true loss of efficiency over time occurred. This investigation provides new insight of the mechanisms underpinning VO_2sc_ and exercise tolerance.

The fitness level of our sample measured during the ramp incremental tests indicated that the participants of this investigation were active subjects. (VO_2peak_: 49 ± 3 ml*min^−1^*kg, see Table 1 for the other characteristics) [1], and presented an overall metabolic response to constant load exercise consistent with previous studies (e.g. no VO_2sc_ in the moderate domain; VO_2sc_ in heavy +4.2 ± 3.4% tending to steady-state; VO_2sc_ in severe domain +11.4 ± 4.9% tending to VO_2max_) [17, 25, 29].

By applying an approach previously proposed by O’Connell et al. [17], our study quantified the individual contributors to the overall net VO_2_ cost of exercise (i.e. AdjO_2Eq_) and their interplay during the metabolic transition and steady-state. While O’Connell et al. studied the VO_2_ of active muscles, the VO_2_ associated with ventilation and the VO_2_ equivalent of [La^−^] in the severe domain only, we extended the analysis to the three exercise intensity domains.

As expected, based on the changes in ventilation described above, the VO_2_ associated with ventilation increased going from moderate (3 ± 3% of the VO_2sc_), heavy (7 ± 3%), to severe (14 ± 7%) intensity. In agreement with previous work [20], this finding confirms that ~ 85% of the VO_2sc_ occurs within the working muscles. In addition, our study demonstrated that the VO_2_ associated with ventilation increased over time in the severe domain only. Furthermore, for all the exercise domains, the O_2_ equivalent of [La^−^] accumulation decreased after the transition phase (i.e. 0-to-3-time segment), compatible with a progressive decrease of the contribution of glycolysis to ATP resynthesis over time [22]. Interestingly, in the domains below RCP, this decreased contribution of glycolysis to the energy provision was “mirrored” by an increased contribution of oxidative metabolism, to satisfy an invariant energetic demand (Table 3 and Fig. 3). In other words, the VO_2sc_ that is observed in the heavy domain may be related to a prolonged “metabolic shift” between anaerobic and aerobic energy sources of ATP resynthesis rather than to an augmented cost of locomotion over time. Such a view is in agreement with the findings and interpretations of previous human studies, performed in the heavy/severe domain of exercise [16, 17] as well as with studies based on computer modelling of the skeletal muscle bioenergetics [15]. For the exercise in the severe domain, however, we found that accounting for the total energetic cost of locomotion did not completely explain VO_2sc_. We were the first to demonstrate that, in this domain, the AdjO_2Eq_ continued to increase showing an augment energy required to sustain the same PO, compatible with a true loss of efficiency over time.

Relative to the severe intensity domain, the partial discrepancy between our data and O’Connell’s may be explained by methodological differences between both studies: (i) as we did, most studies in the field of VO_2_ kinetics define intensity domains based on gas exchange thresholds [10, 23]; on the contrary, O’Connell used lactate-based measures. Yet, the correspondence between the above methods remains controversial [3, 12, 13] and the difference may have led to unmatched intensity domains among our studies. (ii) O’Connell incremental protocol for VO_2peak_ detection was performed after 10 min of recovery from a ~ 20-min protocol with 3-min steps used for LT determination. This approach could be responsible for an underestimation of peak-PO and consequently of ∆60% and may not have guaranteed an exercise intensity corresponding to the severe domain for all participants. In summary, while a direct comparison between different studies may be difficult, our data provides the first, comprehensive, domain-specific characterization of the contributors to the observed VO_2sc_.

Traditionally, VO_2sc_ has been attributed to an increased cost of locomotion when exercise is protracted more than 3 min at a constant workload in the heavy and severe exercise intensity domains, in relation to either fatigue or recruitment of higher-order motor units or both [9]. Interestingly, while a clear distinction between exercises performed above and below RCP is a common concept in exercise physiology [10], VO_2sc_ measured in the heavy and in the severe exercise domains is usually considered as the expression of a single phenomenon [7, 9]. In this context, a recent paper based on mathematical modelling of the muscle bioenergetics, proposed that a metabolic shift between the aerobic and the anaerobic energy systems, caused by a progressive inhibition of the glycolytic ATP supply by cytosol acidification, may also contribute to VO_2sc_ [15]. The authors also suggest that the size of the VO_2sc_ can increase when the contribution of glycolysis to ATP resynthesis (and in turn proton accumulation) is higher; a lower VO_2_ in the initial stages of exercise rather than an increased VO_2_ after 3–6 min into exercise may explain the larger VO_2sc_ at the higher exercise intensities [15].

This is the first experimental study to determine the contributors of VO_2sc_ in the three intensity domains of exercise. Our findings (i.e. a prolonged metabolic shift in the heavy domain and a true loss of efficiency over time in the severe intensity domain only) appear to support the theory proposed by Korzeniewski & Zoladz in 2015. We speculate that, with increasing exercise intensity, the recruitment of bigger, preferentially glycolytic muscle fibres could explain a higher contribution of glycolysis to ATP resynthesis at exercise onset and therefore a delayed VO_2_ steady state. Furthermore, the recruitment of these intrinsically less efficient and fatigable type II fibres could explain the true loss of efficiency that appears over time in the severe domain of exercise [5, 7, 9]. The novelty and the practical implications of this, if confirmed by further studies, would be: (i) in the heavy domain, the VO_2sc_ is the expression of a delayed adjustment of VO_2_ rather than of a loss of efficiency developing over time; (ii) contrary to what is currently accepted, the adjustment of VO_2_ in the heavy domain may be described by a slow primary component rather than by the summation of a primary plus a slow component; (iii) in the severe domain the VO_2sc_ may be explained by both a prolonged metabolic shift and a true loss of efficiency over time; (iv) VO_2sc_ likely has different physiological underpinnings in the heavy and severe domain of exercise; therefore, we should be mindful of this when the two intensity paradigms are used in the context of VO_2sc_ as different interventions may produce different effects in the two domains.

In conclusion, the innovative methodological approach applied in this study allowed to discriminate three contributors to the VO_2sc_: increased VO_2_ cost of ventilation, prolonged shifting between aerobic and glycolytic metabolism, and loss of efficiency over time. How these mechanisms contribute to the VO_2sc_ depends on relative exercise intensity, with a true loss of efficiency over time occurring only in the severe domain.

## Limitations

It should be acknowledged that data interpretation in this investigation depends upon estimates of the energetic yield of lactate accumulation and of the VO_2_ cost of ventilation and ignores the contribution of oxygen stores and anaerobic alactic mechanism of ATP resynthesis. These estimates rely on some assumptions (e.g. fingertip capillary lactate reflects whole-body lactate accumulation) and systematic biases in applying the same values/equations to different subjects. In the belief that these limitations should not have altered the interpretation of our results; we provided a deep discussion on these issues for the interested reader as supplementary material of this article.

## Electronic supplementary material

ESM 1 (DOCX 18 kb)
