# Supplementary material for: Bioenergetics of the VO2 slow component between exercise intensity domains
Source: Pflugers Arch. 2020 Jul 14;472(10):1447–56. doi: 10.1007/s00424-020-02437-7 (PMC7476983; doi:10.1007/s00424-020-02437-7)
Supplement: Supplementary file 1 — (DOCX 18 kb) [file 424_2020_2437_MOESM1_ESM.docx]

**Limitations:**

It should be acknowledged that data interpretation in this investigation depends upon estimates of the energetic yield of lactate accumulation and of the VO_2_ cost of ventilation and ignores the contribution of oxygen stores and anaerobic alactic mechanism of ATP resynthesis.

The di Prampero equation [25], was used to obtain the energy equivalent of blood lactate accumulation. This equation was developed using the following approach: first the O_2_ cost of a supramaximal exercise was estimated by extrapolating the sub-maximal VO_2_/PO relationship; then, the difference between the estimated and the actual VO_2_ of exercise was calculated; finally, this difference was divided by the lactate accumulated over the time of the measure in order to calculate the O_2_ equivalent of lactate accumulation.

The above described method relies on the following assumptions: *i)* fingertip capillary lactate reflects whole-body lactate accumulation; *ii)* blood lactate accumulation in *whole-body, in vivo conditions* reflects the net result of whole-body lactate production and clearance and therefore it is an indicator of the net glycolytic contribution to ATP resynthesis. This approach has been equally criticized and endorsed and its validity is still debated. Clearly, using the same fixed value of O_2_ equivalent of lactate accumulation for all the individuals may impact the accuracy of the estimate of the glycolytic contribution to exercise at the individual level. However, this systematic bias would have a similar impact across domains and across time, and therefore should not preclude our ability to estimate the contribution of anaerobic metabolism to the overall cost of exercises in our experimental setup. An extensive explanation of the advantages and the limitations of this method is reported elsewhere [25].

Referring the VO_2_ cost of ventilation, different predictive equations were proposed for calculating the work of ventilation and its translation in VO_2_ cost [1, 2, 7] and a variability across individuals of around ±10% has been described [1, 2]. With the aim to quantify the effect of using different predictive equations, we compared the equations from Aaron et al. (characterized by a higher cost of ventilation) and the equations of Coast et al. (used in our study) at different levels of ventilation. Using Aaron’s compared to Coast’s equation would cause an increase in the estimated cost of ventilation from a minimum of 19% in the moderate to a maximum of 25% in the severe domain of exercise. The effect of using the less conservative Aaron’s equation would be a larger contribution of ventilation to the VO_2sc_, implying a smaller loss of efficiency over time for all exercise intensities. Finally, as also discussed in relation to the O_2_ equivalent of lactate, the use of an identical equation for different individuals may have affected the accuracy of our calculations; however, this systematic bias would have a similar impact across domains and across time, and therefore should not preclude our ability to estimate the contribution of the cost of ventilation to the overall cost of exercises in our experimental setup. As a final consideration, a possible effect of time or fatigue on the VO_2_ cost of ventilation should also be considered. However, to our knowledge the existence of this phenomenon, its temporal appearance and its magnitude have not yet been described. A reduction of the VO_2_ cost of ventilation over time during a constant load exercise appears very unlikely. On the contrary, similarly to the loss of efficiency of locomotor muscles, a loss of efficiency of ventilator muscles might manifest when exercise is protracted. Also in this case, the effect of an unaccounted loss of efficiency of ventilation over time on our results would imply an overestimation of the locomotor loss of efficiency.

Finally, during the first three minutes of exercise, the contribution of the immediate energy sources (i.e. the O_2_ stores and phosphocreatine contribution) to ATP resynthesis were not quantified. Ignoring this contribution, quantifiable around 680±90 ml of O_2_ [11, 25], has clearly caused an underestimation of the energy cost of exercise during the onset phase, but should not have altered the cost quantification of the following part of exercise, and therefore the interpretation of our findings in the relation to the VO_2sc_.
